# Effect of Sputtering Power on the Microstructure and Tribological Properties of TiN/TiAlN Coatings Prepared by DC Magnetron Sputtering

**DOI:** 10.3390/ma19132742

**Published:** 2026-06-26

**Authors:** Haochen Zhang, Huiwei Du, Jiaqin Li, Youfa Yu, Jiangying Wang

**Affiliations:** 1 School of Materials and Chemistry, China Jiliang University, Hangzhou 310018, China; p23050854058@cjlu.edu.cn; 2 Zhejiang Brilliant Refrigeration Equipment Co., Ltd., Shaoxing 312500, China; jiaqinli@outlook.com (J.L.); wangshaobo@liyongda.com (Y.Y.); 3 Xinchang China Jiliang University Enterprise Innovation Research Institute Co., Ltd., Shaoxing 312500, China

**Keywords:** steel substrate, TiN/TiAlN coatings, DC magnetron sputtering, sputtering power, microstructure, tribological properties, wear resistance

## Abstract

**Highlights:**

- TiN/TiAlN coatings were deposited on steel substrates by DC magnetron sputtering.
- Sputtering power affected coating morphology, phase structure, adhesion and wear behavior.
- The coating deposited at 140 W showed the lowest measured average friction coefficient and specific wear rate within the tested coating series.
- A moderate sputtering power was associated with coating densification and higher measured adhesion.
- Excessive sputtering power was associated with particle coarsening and poorer wear resistance.
- In the present sample series, 140 W provided a useful processing window for coating densification, adhesion and wear resistance.

**Abstract:**

TiN/TiAlN coatings were deposited on 40Cr steel substrates by DC magnetron sputtering to improve the surface tribological performance of the steel. The influence of sputtering power (80, 100, 120, 140, 160 and 180 W) on coating morphology, phase structure, adhesion strength and wear behavior was evaluated using SEM, EDS, XRD, Vickers microhardness testing, scratch testing and ball-on-disk tribological testing. The coatings were dense and relatively smooth, with only a small number of submicron particles. Increasing sputtering power increased the coating thickness, and the EDS results suggested an increase in Al content of up to 160 W, whereas the crystallite size of the TiAlN (200) phase first decreased and then increased. XRD analysis showed that the coatings were dominated by face-centered cubic TiAlN, accompanied by weak TiN and AlN diffraction peaks. Among the tested samples, the coating deposited at 140 W showed the most favorable measured combination of adhesion and tribological properties within the tested series, with a thickness of 1.76 μm, a Vickers microhardness of 906.35 HV0.25, an adhesion strength of 45.6 N, an average friction coefficient of 0.322 and a specific wear rate of 28.37 × 10^−7^ mm^3^ N^−1^ m^−1^. These measured trends are consistent with the dense morphology, refined crystallites, high microhardness and higher measured adhesion observed at moderate sputtering power. In contrast, excessive sputtering power was associated with particle coarsening and coating defects, accompanied by higher measured friction and wear.

## 1. Introduction

Medium-carbon quenched-and-tempered steels are widely used in rotors, shafts, gears, molds and other key components of screw refrigeration compressors. During service, these steel components experience repeated friction and wear, which can reduce machining accuracy, reliability and service life. In this study, 40Cr steel was selected as the substrate to evaluate TiN/TiAlN protective coatings under a representative wear-prone steel surface. Surface modification with hard protective coatings is therefore an effective route for improving the tribological performance of such steel substrates. TiN hard coatings have been widely used to reduce friction and adhesive wear on metallic surfaces; however, TiN can oxidize to TiO_2_ at elevated temperatures, limiting its long-term stability under oxidative service conditions [1,2].

Elemental alloying and stacked coating design are widely used to improve TiN-based coatings. Incorporating Al, Zr, Si or Cr can enhance oxidation resistance and mechanical performance [3,4,5,6,7]. Among these elements, Al is particularly attractive because Ti_1−x_Al_x_N can form a NaCl-type solid solution in which Al is incorporated into the TiN lattice [8]. Reviews and experimental studies of (Ti,Al)N coatings show that Al addition and stacked or layered coating configurations can improve hardness, oxidation resistance and wear resistance by modifying phase stability and nanoscale microstructure [9,10]. However, thermal-expansion mismatch between TiAlN coatings and 40Cr steel may contribute to residual stress at the coating/substrate interface, while unfavorable deposition conditions can promote coarse grains and reduce coating adhesion [11]. A nominal Ti transition/TiN/TiAlN stacked coating may therefore help improve coating support and adhesion stability compared with single-layer TiN or TiAlN coatings [12].

TiN/TiAlN coatings can be prepared by ion-beam sputtering, magnetron sputtering, arc ion plating and cathodic arc evaporation. Magnetron sputtering is attractive because it enables controllable deposition, uniform thickness and smooth, dense coatings over relatively large surface areas [13]. Previous studies have shown that sputtered Ti/TiN bilayers and TiN/TiAlN coatings are sensitive to deposition conditions, including substrate state, target power, temperature and substrate bias, which collectively affect film structure, adhesion and service performance [14,15,16]. Nevertheless, the coupled influence of sputtering power on the microstructure, adhesion strength and tribological behavior of TiN/TiAlN coatings on 40Cr steel under low-temperature deposition conditions remains insufficiently clarified. Here, TiN/TiAlN coatings were deposited on 40Cr steel substrates by DC magnetron sputtering, and the effect of sputtering power from 80 to 180 W on coating structure, adhesion and wear behavior was systematically investigated.

## 2. Materials and Methods

**Figure 1:**
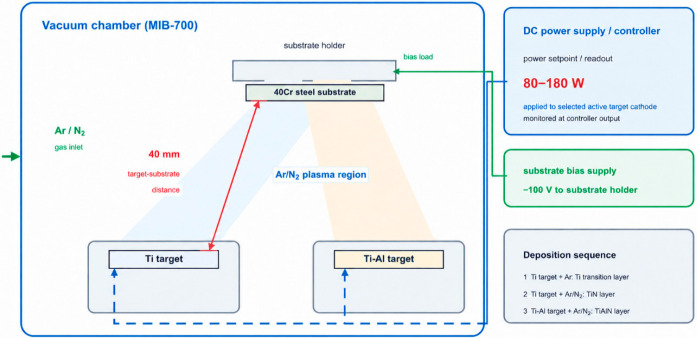
Schematic diagram of the DC magnetron sputtering configuration and nominal Ti transition/TiN/TiAlN stacked coating deposition. The Ti target was used for the Ti transition and TiN layers, the Ti-Al target was used for the TiAlN layer, and the sputtering power was applied to the selected active target cathode and monitored by the DC power supply/controller. Schematic not to scale.

Cylindrical 40Cr steel specimens with a diameter of 20 mm and a thickness of 5 mm were used as substrates. TiN/TiAlN coatings were deposited using TiN/TiAlN coatings were deposited using a custom-built MIB-700 multifunctional ion-beam-assisted sputtering system at China Jiliang University (Hangzhou, China). The sputtering targets were a Ti-Al alloy target with a Ti:Al atomic ratio of 1:1 and a purity of 99.5%, and a Ti target with a purity of 99.99%. The target–substrate distance was 40 mm, and the Ti-Al target size was 60 mm × 3 mm. The deposition configuration and nominal layer sequence are shown schematically in Figure 1. During deposition, the sputtering power (80–180 W) was applied to the selected active target cathode and was set and monitored by the DC power supply/controller of the MIB-700 system, while the substrate was maintained at a bias voltage of −100 V. Only one cathode was energized at a time during each deposition step.

Before deposition, the substrates were ground successively with 400-, 800-, 1200- and 1600-mesh SiC abrasive papers and then polished with a polishing cloth. The polished substrates were ultrasonically cleaned alternately in acetone and ethanol for 10 min to remove polishing residues and surface oil. The chamber was evacuated to a base pressure below 1.5 × 10^−3^ Pa. High-purity Ar (99.99%) was then introduced at 5 sccm, and, for each sample, a Ti transition layer was sputtered from the Ti target for 10 min at a working pressure of 0.5 Pa under the corresponding selected sputtering-power condition. High-purity N_2_ (99.99%) was subsequently introduced at 5 sccm. The N_2_:Ar flow ratio was maintained at 1:1, and the working pressure was stabilized at 1 Pa. For each sample, the same selected nominal sputtering power was used during the Ti transition, TiN and TiAlN deposition steps. The TiN layer was deposited from the Ti target for 40 min under the Ar/N_2_ mixed atmosphere, followed by deposition of the TiAlN layer from the Ti-Al target for 40 min under the same selected nominal sputtering-power condition. Thus, the deposited coating is described as a nominal Ti transition/TiN/TiAlN stacked coating on the 40Cr steel substrate. This description denotes the intended deposition sequence rather than direct high-resolution confirmation of periodic individual layers. The individual sublayer thicknesses could not be separately resolved from the present cross-sectional SEM images; therefore, the deposition times for each layer and the measured total coating thicknesses are reported instead, without assigning unsupported individual sublayer thickness values. The substrate bias voltage was −100 V, and deposition was performed at room temperature.

Surface and cross-sectional morphologies were examined using a Phenom ProX scanning electron microscope (Phenom-World/Thermo Fisher Scientific, Eindhoven, The Netherlands).

Elemental composition was analyzed using the energy-dispersive spectroscopy (EDS) detector attached to the same Phenom ProX SEM.

Because EDS has limited accuracy for light elements such as nitrogen, the elemental compositions are used mainly for semi-quantitative comparison among coatings prepared at different sputtering powers, rather than for exact stoichiometric determination.

Phase composition was characterized using a TD-3500 X-ray diffractometer (Dandong Tongda Science & Technology Co., Ltd., Dandong, China), and the diffraction peaks were compared with standard reference cards.

Coating adhesion was evaluated using a WS-2005 automatic scratch tester (Lanzhou Zhongke Kaihua Technology Development Co., Ltd., Lanzhou, China) with a scratch length of 4 mm and a maximum applied load of 60 N.

Vickers microhardness was measured using an HVS-1000 microhardness tester (Shanghai Zhongyan Instrument Manufacturing Co., Ltd., Shanghai, China), and the coating hardness values are reported as HV0.25.

Tribological properties were evaluated using a WTM-2E micro-tribometer (Lanzhou Zhongke Kaihua Technology Development Co., Ltd., Lanzhou, China).

The tests were conducted in ambient laboratory air at room temperature; relative humidity was not controlled or recorded, and no controlled gas atmosphere was introduced. Si_3_N_4_ ceramic balls with a diameter of 4 mm were used as the counterface. The test duration was 10 min, the rotation speed was 500 r/min, the applied load was 0.98 N and the rotation radius was 2 mm, corresponding to a sliding speed of approximately 0.105 m s^−1^ and a total sliding distance of approximately 62.8 m.

After wear testing, the wear-scar morphology was analyzed using a VHX-700FC three-dimensional microscope (KEYENCE Corporation, Osaka, Japan).

The specific wear rate, *K*, which is also referred to as the wear factor in tribological testing [17], was calculated as follows:

(1) $$K\;=\;\frac{W_{v}}{F\times L}$$

(2) $$W_{v}=\frac{2\pi hr}{6b}(3h^{2}+4b^{2})$$

(3) $$L=\frac{2\pi nrt}{1000}$$

where *K* is the specific wear rate (mm^3^ N^−1^ m^−1^), *W_v_* is the wear volume of the specimen (mm^3^), *F* is the applied load (N), *L* is the relative sliding distance (m), *h* is the wear depth (mm), *b* is the wear width (mm), *n* is the rotation speed (r/min), *r* is the rotation radius of the ball (mm) and *t* is the test time (min). In Equation (3), *r* is expressed in mm, and the factor 1000 converts the sliding distance from mm to m. Complete replicate datasets were not available for all performance metrics in the present study; therefore, standard deviations and confidence intervals are not reported. For adhesion strength, friction coefficient, wear volume and specific wear rate, one representative test result was available for each sputtering-power condition. Here, *n* = 1 denotes one available result for a given coating condition and does not imply that the measured value is error-free. Rather, it indicates that random scatter among independent replicate tests could not be estimated from the present dataset. Therefore, the performance differences among coatings are discussed as experimental trends within the tested sample series rather than as statistically ranked differences.

## 3. Results and Discussion

**Figure 2:**
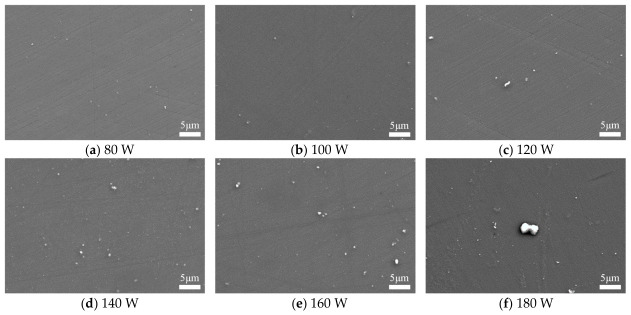
Surface morphologies of TiN/TiAlN coatings prepared at different sputtering powers.

Figure 2 shows the surface morphologies of TiN/TiAlN coatings deposited at different sputtering powers. All coatings exhibit dense and smooth surfaces without obvious cracks or large defects. Only a small number of submicron particles are observed. As the sputtering power increases, both the number and size of surface particles gradually increase, which may increase surface roughness and consequently affect the friction coefficient [18].

**Figure 3:**
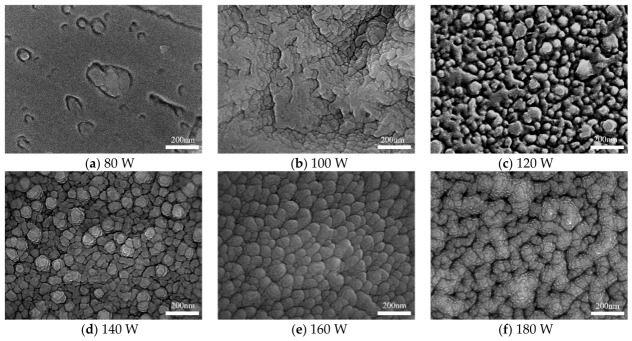
Surface grain morphologies of TiN/TiAlN coatings prepared at different sputtering powers.

Figure 3 presents the surface grain morphologies of the coatings. At 80 and 100 W, the grains are relatively incomplete, which may be associated with insufficient particle energy and slow grain growth. At 120 W, cellular grains begin to form, although visible gaps remain between grains. At 140 W, the coating exhibits a denser structure with finer and more uniform grains. Further increasing the sputtering power to 160 and 180 W produces larger grains and more obvious intergranular spacing, which may result from an excessive deposition rate and enhanced grain growth at high sputtering power.

**Figure 4:**
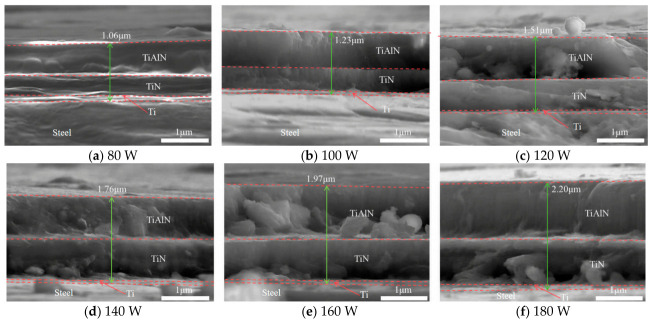
Cross-sectional morphologies of TiN/TiAlN coatings prepared at different sputtering powers. The dashed lines indicate the nominal layer sequence according to the deposition process, from bottom to top: 40Cr steel substrate, Ti transition layer, TiN layer and TiAlN layer. The vertical green double-headed arrows indicate the measured total coating thickness of the nominal stacked coating. Individual sublayer thicknesses could not be reliably resolved from the present cross-sectional SEM images.

Figure 4 shows the cross-sectional morphologies of the coatings. Coating thickness increases with increasing sputtering power because higher power enhances target ionization and sputtering yield, allowing more Ti, Al and N species to be deposited on the substrate within the same deposition time. The measured coating thicknesses are approximately 1.06, 1.23, 1.51, 1.76, 1.97 and 2.20 μm for the coatings deposited at 80, 100, 120, 140, 160 and 180 W, respectively. Overall, the measured total coating thickness ranged from 1.06 to 2.20 μm depending on sputtering power. These values correspond to the total coating thickness of the nominal Ti transition/TiN/TiAlN stacked coating, rather than the individual sublayer thicknesses. The increased particle energy may also promote diffusion and reaction at the growing film surface. The elemental compositions of the coatings deposited at different sputtering powers are listed in Table 1.

**Figure 5:**
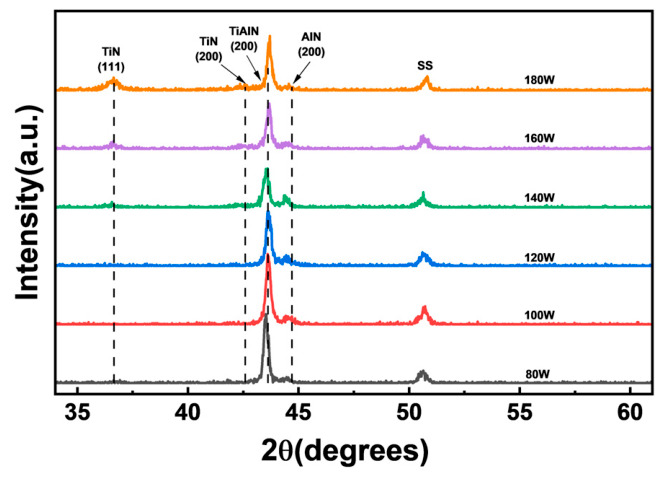
XRD patterns of TiN/TiAlN coatings prepared at different sputtering powers.

As shown in Figure 5, the coatings deposited at different sputtering powers are mainly composed of a TiAlN phase with a face-centered cubic (fcc) structure. Weak diffraction peaks corresponding to TiN (111), TiN (200) (PDF #38-1420) and AlN (200) (PDF #46-1200) are also detected, indicating a multiphase structure dominated by TiAlN with small amounts of TiN and AlN. The TiN (111) peak becomes visible at sputtering powers of 140–180 W. As sputtering power increases, the intensity of the TiAlN (200) peak first decreases and then increases, whereas the AlN (200) peak first increases and then decreases. This intensity variation is most pronounced at 140 W. Because temperature, gas flux, diffusion conditions and plasma parameters can influence phase formation during thin-film deposition [13,19], the observed changes in diffraction intensity are consistent with changes in deposition energy and growth kinetics at different sputtering powers. The variation in the full width at half maximum (FWHM) of the TiAlN (200) peak is interpreted in terms of crystallite size and possible lattice-strain contributions to XRD line broadening [20].

Table 2 summarizes the FWHM and crystallite size calculated from the TiAlN (200) diffraction peak. With increasing sputtering power, the FWHM increases from 0.238° to 0.294° and then decreases to 0.250°, while the XRD-derived crystallite size decreases from 39.5 nm to 30.9 nm and then increases to 37.4 nm. This trend agrees with reports that increasing magnetron discharge power can refine crystallites in sputtered titanium aluminium nitride coatings under suitable growth conditions [21]. The coating deposited at 140 W has the smallest XRD-derived crystallite size of 30.9 ± 0.4 nm, which represents coherent diffraction domains rather than the larger surface features observed by SEM. Together with the compact morphology shown in Figure 3, this result is consistent with greater coating densification at moderate sputtering power. Dense TiN/TiAlN coating structures have been associated with improved mechanical and tribological performance [22], although the present study does not claim direct high-resolution confirmation of periodic layer-by-layer stacking.

**Figure 6:**
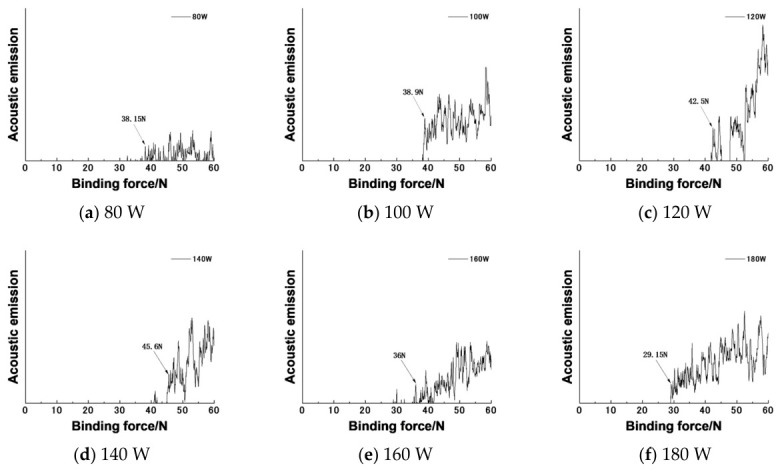
Scratch adhesion test results of TiN/TiAlN coatings prepared at different sputtering powers.

Figure 6 shows that the adhesion strength of TiN/TiAlN coatings first increases and then decreases with increasing sputtering power. The highest measured adhesion strength of 45.6 N was obtained at 140 W. From 80 to 140 W, increasing sputtering power likely increases the flux and energy of deposited species, which may promote surface mobility, coating densification and interfacial bonding [19]. These effects are consistent with the higher measured adhesion. When the sputtering power exceeds 140 W, larger surface particles, coarser grains and potentially higher residual stresses can develop. Excessive residual stress in thin hard coatings can promote fracture and delamination, thereby reducing adhesion strength [23].

Vickers microhardness measurements provide additional support for the mechanical response of the coatings. The coating hardness increased from 430.2 HV0.25 at 80 W to a maximum of 906.35 HV0.25 at 140 W and then decreased to 830.92 HV0.25 at 180 W. This trend is consistent with the refined crystallites and dense morphology obtained at moderate sputtering power and with the coarser grains and surface defects formed at excessive power. The high measured hardness of the 140 W coating, together with its maximum measured adhesion strength, is consistent with greater resistance to local plastic deformation during sliding. However, because elastic modulus and H/E-related parameters were not measured, load-bearing behavior cannot be fully quantified and is discussed only on the basis of the combined hardness, adhesion and wear-track evidence.

**Figure 7:**
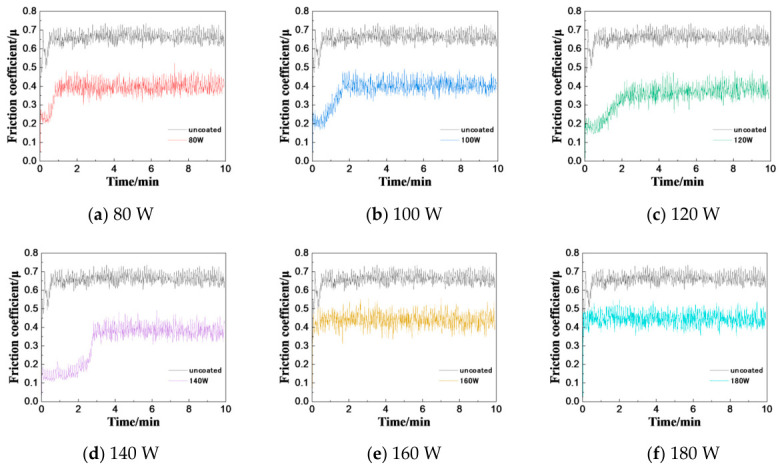
Friction coefficient curves of the uncoated 40Cr steel substrate and TiN/TiAlN coatings prepared at different sputtering powers.

Figure 7 shows the friction-coefficient curves of the 40Cr steel substrate and TiN/TiAlN coatings. The average friction coefficient of the uncoated substrate is 0.657, whereas those of the coatings deposited at 80, 100, 120, 140, 160 and 180 W are 0.386, 0.381, 0.343, 0.322, 0.438 and 0.445, respectively. Thus, all coatings reduce the friction coefficient relative to the substrate. After an initial running-in period, the curves gradually approach quasi-steady values; however, the transition duration varies with sputtering power, and the coatings deposited at 120 and 140 W require a longer transition before reaching a relatively stable friction state. During running-in, fine surface particles may increase roughness and cause fluctuations in the friction curve. As sliding continues, loose particles are gradually removed, and the friction coefficient becomes more stable. Overall, the friction coefficient first decreases and then increases with increasing sputtering power. The reduced friction at moderate power is consistent with a denser coating structure, suitable coating thickness, higher microhardness and higher measured adhesion; however, because elastic modulus and H/E-related parameters were not measured, this relationship is interpreted as an experimental trend rather than as a direct quantification of load-bearing behavior. In contrast, the higher friction coefficients at 160 and 180 W are associated with larger particles and increased surface roughness produced under excessive sputtering power.

**Figure 8:**
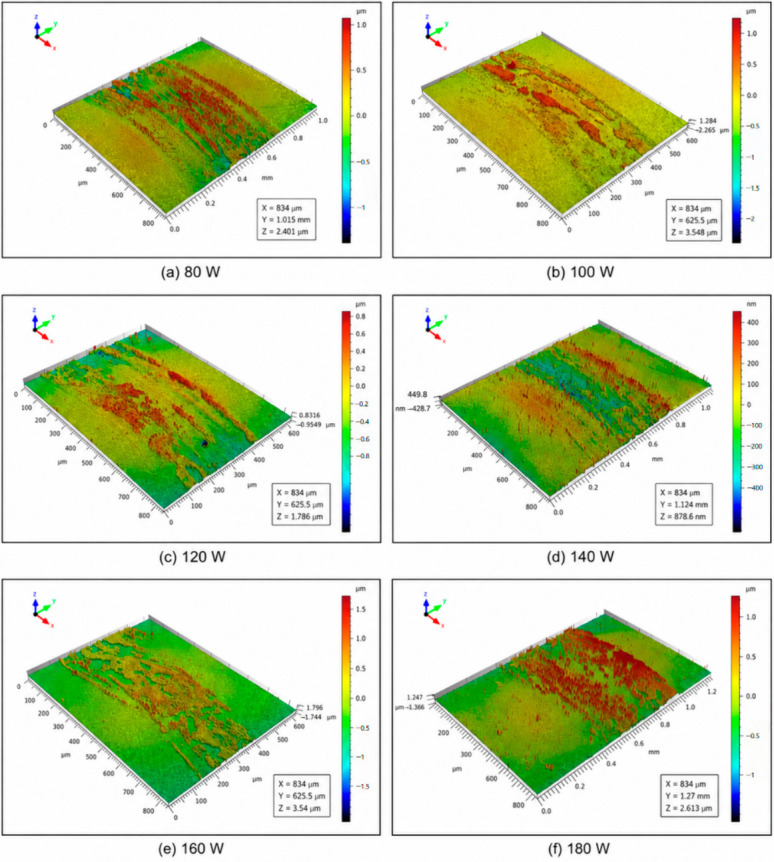
Three-dimensional wear-scar morphologies of TiN/TiAlN coatings prepared at different sputtering powers.

The three-dimensional wear-scar morphologies are shown in Figure 8. The average wear-scar depths of coatings prepared at 80, 100, 120, 140, 160 and 180 W are 0.2345, 0.0785, 0.0924, 0.1390, 0.1098 and 0.1183 μm, respectively, and the corresponding wear-scar widths are 0.26, 0.33, 0.29, 0.15, 0.25 and 0.40 mm, respectively. Although the wear-scar depth does not vary monotonically with sputtering power, the coating deposited at 140 W exhibits the narrowest wear scar and the lowest calculated wear volume (Table 3), consistent with favorable wear resistance within the tested series. At lower sputtering powers, insufficient particle energy and relatively poor coating densification may contribute to more severe wear damage, whereas the broad wear scar at 180 W is consistent with defect formation under excessive sputtering power. These trends are consistent with structure-zone and film-growth models for sputter-deposited films [19,24], and target-power-dependent changes in film structure and tribological response have also been reported for sputtered composite films [25].

Table 3 lists the wear volume and specific wear rate of the coatings. The coating deposited at 140 W exhibits the lowest measured wear volume and specific wear rate, indicating favorable wear resistance within the tested sputtering-power series. Because complete replicate datasets and standard deviations are not available for all performance metrics in the present study, the differences among coatings are discussed as experimental trends within the tested sample series rather than as statistically ranked differences. Future work will include systematic repeated tests and statistical analysis to further verify the reproducibility of the observed trends.

**Figure 9:**
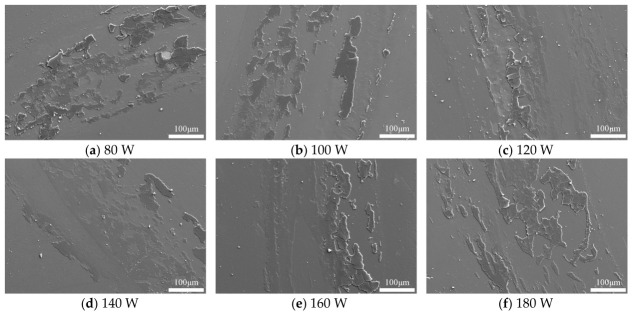
Worn surface morphologies of TiN/TiAlN coatings prepared at different sputtering powers.

Figure 9 shows the worn surface morphologies of the coatings. The friction curves in Figure 7 and the worn tracks in Figure 9 are mutually consistent: during the initial running-in period, fluctuations in the friction coefficient can be associated with the removal and redistribution of loose particles generated from the coating surface. Grooves and abrasive debris are observed on the worn surfaces, suggesting that abrasive wear contributes during sliding. Local accumulation of debris and coating material suggests that adhesive wear may also contribute [26]. Because counterface scars and isolated wear debris were not separately characterized in this study, the wear mechanism is interpreted conservatively from the coating wear tracks and friction curves.

Obvious furrows are observed in Figure 9a,b,f. For the coatings deposited at 80 and 100 W, the relatively thin and less dense coating structure may be less effective in resisting wear deformation. For the coating deposited at 180 W, reduced adhesion and increased defects are consistent with local coating damage under load. In contrast, the coatings deposited at 120, 140 and 160 W show grooves and abrasive debris but no severe furrowing. The coating deposited at 140 W combines refined crystallites, a dense morphology and sufficient total coating thickness, which is consistent with its lower friction coefficient and specific wear rate within the limits of the available wear-track evidence.

## 4. Conclusions

TiN/TiAlN coatings were deposited on 40Cr steel by DC magnetron sputtering, and the influence of sputtering power on coating microstructure, adhesion strength and tribological properties was investigated. The main conclusions are as follows:

(1) TiN/TiAlN coatings prepared at different sputtering powers are dense and relatively smooth, with only a small number of surface particles. Increasing sputtering power increases coating thickness and changes grain morphology, whereas excessive power was associated with larger particles and coarser grains.
(2) The coatings are mainly composed of fcc TiAlN, with weak TiN and AlN diffraction peaks. The crystallite size first decreases and then increases with sputtering power, and the smallest crystallite size of 30.9 ± 0.4 nm is obtained at 140 W.
(3) Adhesion strength first increases and then decreases with sputtering power. The coating deposited at 140 W exhibits the highest measured adhesion strength of 45.6 N and the highest measured Vickers microhardness of 906.35 HV0.25.
(4) The TiN/TiAlN coating deposited at 140 W shows the lowest measured average friction coefficient and specific wear rate within the tested sample series, with an average friction coefficient of 0.322 and a specific wear rate of 28.37 × 10^−7^ mm^3^ N^−1^ m^−1^. These measured trends are consistent with its dense microstructure, refined crystallites, high microhardness and the highest measured adhesion strength. Because complete replicate datasets, elastic-modulus data and H/E-related parameters are not available in the present study, these differences are interpreted as experimental trends, and further repeated tests, statistical analysis and mechanical characterization are needed to verify reproducibility and quantify load-bearing behavior.

## Figures and Tables

**Table 1:** Elemental composition of TiN/TiAlN coatings deposited at different sputtering powers.

Sputtering Power (W)	Elemental Composition (at.%)		
	Ti	Al	N
80	31.20	8.34	60.45
100	30.35	11.63	58.03
120	32.27	14.22	53.26
140	28.95	18.41	52.64
160	21.02	23.72	55.26
180	15.45	22.71	61.84

**Table 2:** FWHM and crystallite size of TiN/TiAlN coatings deposited at different sputtering powers.

Sputtering Power (W)	TiAlN (200)	
	FWHM (°)	Crystallite Size (nm)
80	0.238 ± 0.003	39.5 ± 0.7
100	0.264 ± 0.002	35.0 ± 0.4
120	0.280 ± 0.008	32.7 ± 1.2
140	0.294 ± 0.003	30.9 ± 0.4
160	0.272 ± 0.002	33.9 ± 0.3
180	0.250 ± 0.003	37.4 ± 0.5

**Table 3:** Wear volume and specific wear rate of TiN/TiAlN coatings deposited at different sputtering powers.

Sputtering Power (W)	Wear Volume, Wv (×10^−5^ mm^3^)	Specific Wear Rate, K (×10^−7^ mm^3^ N^−1^ m^−1^)
80	51.08	82.95
100	21.69	35.23
120	22.44	36.45
140	17.47	28.37
160	23.00	37.35
180	39.64	64.38

## Data Availability

The raw data supporting the conclusions of this article will be made available by the authors on request.

## Abbreviations

The following abbreviations are used in this manuscript:

DC	Direct current
SEM	Scanning electron microscopy
EDS	Energy-dispersive spectroscopy
XRD	X-ray diffraction
FWHM	Full width at half maximum
fcc	Face-centered cubic
